# A case (report) for mechanistic validation of meningioma molecular therapies

**DOI:** 10.1093/noajnl/vdac162

**Published:** 2022-10-08

**Authors:** Minh P Nguyen, Kyounghee Seo, Charlotte D Eaton, Calixto-Hope G Lucas, William C Chen, Abrar Choudhury, Jacob S Young, David R Raleigh

**Affiliations:** Department of Radiation Oncology, University of California San Francisco, San Francisco, California, USA; Department of Neurological Surgery, University of California San Francisco, San Francisco, California, USA; Department of Radiation Oncology, University of California San Francisco, San Francisco, California, USA; Department of Neurological Surgery, University of California San Francisco, San Francisco, California, USA; Department of Radiation Oncology, University of California San Francisco, San Francisco, California, USA; Department of Neurological Surgery, University of California San Francisco, San Francisco, California, USA; Department of Radiation Oncology, University of California San Francisco, San Francisco, California, USA; Department of Neurological Surgery, University of California San Francisco, San Francisco, California, USA; Department of Pathology, University of California San Francisco, San Francisco, California, USA; Department of Radiation Oncology, University of California San Francisco, San Francisco, California, USA; Department of Neurological Surgery, University of California San Francisco, San Francisco, California, USA; Department of Radiation Oncology, University of California San Francisco, San Francisco, California, USA; Department of Neurological Surgery, University of California San Francisco, San Francisco, California, USA; Department of Radiation Oncology, University of California San Francisco, San Francisco, California, USA; Department of Neurological Surgery, University of California San Francisco, San Francisco, California, USA; Department of Radiation Oncology, University of California San Francisco, San Francisco, California, USA; Department of Neurological Surgery, University of California San Francisco, San Francisco, California, USA

The identification of recurring somatic mutations has provided a framework for clinical trials of molecular therapy to treat meningiomas.^1,2^ Nevertheless, preclinical data supporting meningioma molecular therapies are sparse, and most recurring somatic mutations are found in meningioma DNA methylation groups with favorable clinical outcomes.^3^ Meningiomas can encode missense mutations in the Hedgehog pathway activator Smoothened (SMO) and express primary cilia, but meningioma cells do not transduce ciliary Hedgehog signals.^4^ These data suggest SMO antagonists such as vismodegib may not be tractable therapies for meningiomas, and indeed, cancers encoding passenger alterations in the Hedgehog pathway may be accelerated by SMO antagonists.^5^

**Figure 1.**
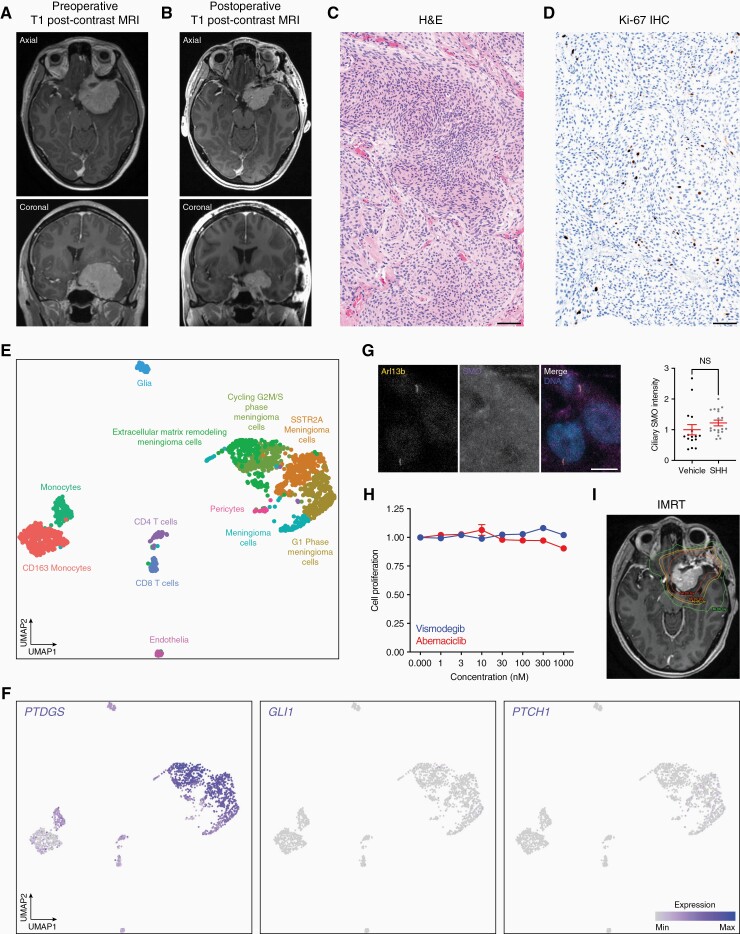
Meningioma and treatment. (A) Preoperative magnetic resonance imaging (MRI). (B) Postoperative magnetic resonance imaging. (C) H&E histology. Scale bar, 100 µm. (D) Ki-67 immunohistochemistry (IHC). Scale bar, 100 µm. (E) Single-cell RNA sequencing UMAP showing cell types distinguished by marker gene expression in accordance with the cellular architecture of human meningiomas.^3^ (F) Single-cell RNA sequencing feature plots showing meningioma marker genes (*PTDGS*) or Hedgehog target genes (*GLI1*, *PTCH1*). (G) SF13398 immunofluorescence and quantification of ciliary SMO intensity after 24 hours of treatment with recombinant Sonic Hedgehog (SHH, 1 µg/mL) compared to vehicle control. Cilia are marked by Arl13b immunofluorescence. Lines show means and error bars show standard error of the mean. NS, nonsignificant (Student’s *t*-test). Scale bar, 10 µm. (H) SF13398 cell proliferation after 5 days of treatment with vismodegib or abemaciclib. Points show means and error bars show standard error of the mean. (I) Intensity modulated radiotherapy (IMRT) isodose lines overlaid on postoperative MRI.

We present the case of a 45-year-old female patient who underwent MRI for subacute left eye vision changes, revealing a dural based, homogenously enhancing lesion arising from the sphenoid wing (Figure 1A). Frontotemporal orbitozygomatic craniotomy was performed for tumor resection, and a postoperative MRI demonstrated residual tumor along the left anterior clinoid, left cavernous sinus, and prepontine cistern (Figure 1B).

Histologic examination showed a solid, moderately to highly cellular neoplasm comprised of spindled to epithelioid cells growing in nests and fascicles (Figure 1C). The tumor cells had round nuclei, variably sized nucleoli, and abundant eosinophilic cytoplasm. Foci of brain invasion, small cell change, hypercellularity, loss of architecture, and areas of necrosis were noted. There were up to 2 mitotic figures per 2 mm^2^ (Figure 1D). These findings were consistent with an atypical meningioma, WHO grade 2.

DNA methylation profiling (https://william-c-chen.shinyapps.io/MeninMethylClassApp/) revealed the meningioma classified as Merlin-intact.^3^ Targeted next generation sequencing of the meningioma revealed a somatic mutation in *SMO*. The mutant allele frequency was 40% of 642 reads and annotated as missense p.L412F (c.1234C>T). *SMO* L412F mutation is associated with Hedgehog pathway misactivation in other cancers,^6^ and is among the most common Hedgehog pathway mutations in meningiomas.^1^ However, *SMO* L412F mutation is also associated with vismodegib resistance,^6^ and does not drive ciliary Hedgehog signaling in meningioma cells.^4^

Single-cell RNA sequencing was performed on 1717 cells from the meningioma. Cell clusters were defined in accordance with the reported cellular architecture of human meningiomas (Figure 1E),^3^ and no evidence of Hedgehog target gene expression was identified (Figure 1F), suggesting the *SMO* L412F mutation was not associated with Hedgehog pathway misactivation. Immunofluorescence of a primary meningioma cell line derived from the resection specimen (SF13398) showed SMO accumulation in primary cilia did not increase in response to recombinant Sonic Hedgehog stimulation (Figure 1G). Treatment of SF13398 cells with vismodegib or the cell cycle inhibitor abemaciclib, which blocks the growth of Immune-enriched or Hypermitotic meningiomas,^3^ did not block Merlin-intact SF13398 cell proliferation (Figure 1H).

In the early postoperative period, the patient developed new onset diplopia that was concerning for tumor progression. In the absence of therapeutic alternatives, she was treated with intensity modulated radiotherapy (Figure 1I), which is associated with high rates of local control but can also cause toxicity. The patient experienced episodes of near fainting preceded by an aura that were concerning for seizure during radiotherapy. Later, she developed central adrenal insufficiency, hypothyroidism secondary to hypothalamic dysfunction, and possible pituitary growth hormone deficiency. The radiotherapy dose to her fornix and left hippocampus raises concern for long-term neurocognitive deficits. She continues to have mild visual impairment, but surveillance MRIs have revealed no evidence of tumor growth 12 months after diagnosis.

Despite ongoing clinical trials of meningioma molecular therapy, limited mechanistic or functional understanding of biological drivers has left many meningioma patients without tractable therapeutic options. In this case, the difficulty in predicting benefit of postoperative radiotherapy, the anticipated short- and long-term toxicities of radiotherapy, the patient’s progressive postoperative neurologic symptoms, and the absence of therapeutic alternatives despite extensive multiplatform molecular analyses underscore the unmet need for new molecular therapies to treat meningiomas.
